# Book Reviews

**Published:** 1900-07

**Authors:** 


### BOOK REVIEWS, PAMPHLETS, EXCHANGES.

### BOOK REVIEWS.

[Contributions solicited for review.]

NORMAL HISTOLOGY. By Edward K. Dunham, Ph.D., M.D., Professor General Pathology, Bacteriology and Hygiene in the University and Bellevue Hospital Medical College, New York. Second edition. Illustrated with 244 engravings. Lea Bros. & Co. New York and Philadelphia. 1900.

In this book the principles of this fundamental branch of medical science are set forth concisely and illustrated with clear drawings of the various kinds of tissue elements. The normal structures of the body are considered in nineteen chapters, to which are added sections on histological technique and practical suggestions for the care and use of the microscope.

The volume is one of 305 pages, well printed and thoroughly llustrated, and will prove a valuable text-book for the student and practical worker in histology.

---

THE INTERNATIONAL MEDICAL ANNUAL AND PRACTITIONERS' INDEX. Eighteenth year. 1900. E. B. Treat & Co., New York. Price $3.00.

This is a book of 660 pages, and represents the work of 41 collaborators, thirty-three of whom are English. The medical and Surgical literature of the past year has been fairly thoroughly sifted and that worthy of presentation finds place in the Annual. If one desires to get the pith of the year's progress very much condensed he may do so by purchasing this book. The modest price is a further recommendation.

The proof-reading is not faultless and the illustrations are few and indifferent, otherwise the book is well gotten up.

THE REFRACTION OF THE EYE. A Manual for Students. By Gustavus Hartridge, F.R.C.S., Senior Surgeon to the Royal Westminster Ophthalmic Hospital, London. Tenth edition, 1900. Illustrated, 12mo. P. Blakiston's Sons & Co., 1012 Walnut street, Philadelphia, Pa. Price $1.50 net.

This well-known and popular little treatise is again out in its tenth edition. This alone speaks for its popularity. This feature is due in a great measure to its simplicity and clearness in the exposition of refraction, a subject so frequently surrounded with scientific terms. The various subjects are illustrated with the histories of cases, and this always simplifies any subject. This brochure of Dr. Hartridge's will always remain popular.

---

A TEXT-BOOK OF DISEASES OF WOMEN. By Chas. B. Penrose, M.D., Ph.D., Professor of Gynecology in the University of Pennsylvania, Surgeon to the Gynecean Hospital, Philadelphia. Illustrated. Third edition. Revised. Philadelphia. W. B. Saunders. 1900.

In this work of 518 pages the study and practice of diseases of women is set forth to meet the requirements of the student of medicine. The conservative and radical treatment of diseases of the female pelvic organs is recommended and explained in detail in the text, and further elucidated by many large and small illustrations. The moderate cost of the book appeals to the medical student, for whom it is primarily intended.

---

THE CLINICAL EXAMINATION OF THE URINE, WITH AN ATLAS OF URINARY DEPOSITS. Including 41 original plates, mostly colored. By Lindley Scott, A.M., M.D. Philadelphia. P. Blakiston's Son & Co. 1012 Walnut St. 1900.

This may probably be termed an edition de luxe. It is one of the handsomest and most effective volumes we have ever seen issued from the medical press. The plates, in size and general appearance of the objects depicted, are almost exact reproductions of nature. The book is certain to prove of the highest value, and its worth can be appreciated at a glance.

